# Myeloperoxidase Levels in Pericardial Fluid Is Independently Associated with Postoperative Atrial Fibrillation after Isolated Coronary Artery Bypass Surgery

**DOI:** 10.3390/jcm11237018

**Published:** 2022-11-28

**Authors:** Yuhua Liu, Yunxiao Yang, Xiubin Yang, Kun Hua

**Affiliations:** Department of Cardiovascular Surgery, Beijing Anzhen Hospital, Capital Medical University, Beijing 100029, China

**Keywords:** intraoperative pericardial fluid, postoperative atrial fibrillation, magnetic Luminex assay, myeloperoxidase, atherosclerotic cardiovascular diseases

## Abstract

Background: Postoperative atrial fibrillation (POAF) is the most common complication after surgery for atherosclerotic cardiovascular disease (ASCVD) and leads to extended hospital stays and increased mortality. Myeloperoxidase (MPO) in postoperative pericardial drainage fluid is associated with an increased risk of POAF; however, the correlations between MPO in intraoperative pericardial fluid and POAF remain largely unknown. The aim of the study was to evaluate whether MPO is associated with POAF. Methods: A total of 97 patients with no history of atrial arrhythmia who had undergone coronary artery bypass surgery (CABG) were identified. We prospectively measured the levels of MPO in intraoperative pericardial fluid and blood using the human magnetic Luminex assay. Then, the occurrence of atrial fibrillation was continuously observed by postoperative ECG and telemetry strips until discharge. Results: Our data showed that POAF occurred in 24 of 97 patients (24.74%). MPO levels in blood were higher in the POAF group than the SR group (*p* = 0.064). Patients with POAF had significantly higher intraoperative pericardial fluid MPO levels than patients who remained in SR (*p* = 0.021). There was no significant correlation between pericardial fluid MPO levels and blood MPO levels (r = −0.47, *p* = 0.770). In a multivariable logistic regression model, pericardial fluid MPO levels were significantly associated with a higher risk of POAF (odds ratio = 1.016, 95% confidence interval, 1.001–1.031; *p* = 0.031). Conclusions: Higher intraoperative pericardial fluid MPO levels are linked with POAF in patients undergoing CABG. This finding provides insight into a possible mechanism of MPO in pericardial fluid increase susceptibility to developing POAF in patients undergoing CABG.

## 1. Introduction

Postoperative atrial fibrillation (POAF) is a common complication, occurring in 20–50% of patients after surgery for ASCVD, 10–30% of patients after noncardiac thoracic surgery, and 5–10% of patients after vascular or large colorectal surgery [1,2,3,4]. POAF typically occurs between days 2 and 4 postoperatively [5,6]. POAF occurrence leads to a significant increase in stroke risk, morbidity, and mortality with a consequent increase in hospitalization time and overall costs [7,8]. However, the predisposing determinants of POAF development after coronary artery bypass surgery (CABG) remain unclear.

The cardiac tissue produces many physiologically active substances, such as cytokines, growth factors, and cardiac hormones. The precise mechanisms of the synthesis, secretion, and metabolism of each substance is not fully understood [9]. Because most substances function locally in either an autocrine or paracrine manner in the heart, it is difficult to detect them in the blood of systemic circulation [10,11]. Accordingly, when the major source of these substances is the heart, not only a step-up of coronary sinus levels, but also pericardial fluid levels should be addressed in future studies for elucidating the potential roles of newly identified substances. Pericardial fluid may contain many unknown pathophysiologically important substances. Further studies are necessary to clarify whether pericardial fluid in patients with various heart diseases is functionally significant. Thus, the analysis of pericardial fluid may provide a new strategy to elucidate the pathophysiology of the heart.

Myeloperoxidase (MPO) is a member of the superfamily of heme peroxidases that is mainly expressed in neutrophils and monocytes. Stimulated neutrophils activate their NADPH oxidase (NOX2) to generate large amounts of superoxide, which acts as a precursor of hydrogen peroxide and other reactive oxygen species (ROS) that are generated by their heme enzyme myeloperoxidase. ROS is involved in inflammatory tissue damage and signaling [12]. A previous study demonstrated that perioperative monocyte activation may not only identify patients at risk for POAF, but also define preventive strategies [13]. Elevated MPO levels are associated with inflammation and increased oxidative stress [14]. MPO may be seen as a mediator or an instrument through which inflammation promotes coronary vascular disease at the molecular and cellular levels. MPO is a crucial prerequisite for structural remodeling of the myocardium, leading to an increased vulnerability to preoperational atrial fibrillation (pre-AF) [15]. Structural remodeling of the atria is important in the pathogenesis of ambulatory AF, and this is likely also important for POAF. In previous studies, it was found that MPO was significantly increased in the postoperative pericardial drainage fluid of patients with POAF; however, the MPO of intraoperative pericardial fluid was not explored [16].

The purpose of this study was to evaluate whether levels of MPO are elevated in patients with POAF and to identify whether the MPO levels were associated with the development of POAF.

## 2. Methods

### 2.1. Study Design and Participants

We conducted a retrospective study using prospectively collected blood and intraoperative pericardial fluid from patients undergoing isolated CABG in Beijing An-Zhen Hospital. The study protocol was approved by the Medicine Ethics Committee of Beijing An-Zhen Hospital and adhered to the Declaration of Helsinki. All the participants provided written informed consent. From February 2022 to October 2022, intraoperative pericardial fluid was collected from isolated first-time CABG. The exclusion criteria were patients with history of arrhythmia, tumors, abnormal thyroid function, currently treated with antiarrhythmic drug, or declined to participate. Participants who underwent redo or emergency operation were also excluded. Finally, 97 patients were included in this study. We prospectively measured the levels of MPO in blood and pericardial fluid using the human magnetic Luminex assay. Then, the occurrence of atrial fibrillation was continuously observed by postoperative ECG and telemetry strips until discharge. Routine biochemical parameters were measured in a biochemical analyzer (Hitachi-7600, Tokyo, Japan) using blinded quality control specimens in the Department of the Biochemical Laboratory at Beijing An-Zhen Hospital.

### 2.2. Collection and Storage of Serum and Pericardial Fluid Samples

Fasting whole blood samples were collected by venipuncture before the cardiac surgery. Blood samples were centrifuged at 3000× *g* for 10 min; then, were immediately transferred into polypropylene tubes and stored at −80 °C until use. The surgical procedure was performed under general anesthesia with single-lumen endotracheal intubation. After the median sternotomy, to optimize the surgical times and avoid confounding factors, all the pericardial fluid samples were obtained by suction with a sterile disposable syringe immediately after the incision of the pericardium before heparin administration. Finally, the sample was placed in a sterile pipe and quickly stored at −80°C until testing occurred. Samples were thawed at 4 °C prior to assay performance. CABG was performed on a beating heart without a cardiopulmonary bypass (CPB). According to the specific surgical procedure, the surgical technique and perioperative management were the same for all the patients.

### 2.3. Luminex Assays

Magnetic Luminex^®^ assays are magnetic bead-based antibody microarrays that can be used to facilitate simultaneous quantitation in a single sample [17]. Previous studies have demonstrated the feasibility of using Luminex to measure cytokines in pericardial fluid [16,18,19]. In the study, we custom-made a Luminex (R&D Systems, Inc., Minneapolis, MN, USA) panel including MPO to assess the impact of pericardial fluid on POAF. In addition, all the pericardial fluid samples were optimally diluted to ensure cytokine levels fell within the dynamic detection range of the assay. According to the manufacturer’s instructions, all the standards and samples were run in duplicate.

### 2.4. Evaluation of POAF

Patients were placed on continuous 24 h cardiac monitoring after surgery until patients were discharged from the hospital, and 12-lead ECGs were obtained to confirm rhythm abnormalities. POAF was defined as a new onset of AF sustained for 30 s or more [20]. The occurrence of the first documented POAF episode was the study end point.

### 2.5. Statistical Analysis

Continuous variables with a normal distribution are presented as the mean ± standard deviation (SD); otherwise, they are presented as the median (interquartile range). A *t*-test and *t*’test were used to analyze normally distributed continuous variables. For the non-normally distributed continuous variables, the Mann–Whitney U test was used. The correlation between serum and pericardial fluid MPO levels was determined with the Spearman correlation coefficient. Categorical variables were compared by using the chi-square test with Yates correction. The risk factors were assessed by using univariate and multivariate logistic regression.

Propensity score matching was applied to achieve balanced exposure groups preoperatively (i.e., minimal confounding), in accordance with the recommendations by Lonjon et al. [21]. A total of 97 patients were selected for the study by matching the most relevant clinical variables, such as age, sex, and possible clinical characteristics, such as body mass index, diabetes mellitus, hypertension, EuroSCORE II, triglycerides, total cholesterol, and other routine biochemical parameters. PSM was performed using the nearest neighbor matching algorithm and a 1:1 ratio. A standardized difference less than 0.1 indicated good balance after PSM. Therefore, there were 20 patients in each group (Table 1). All analyses were performed by SPSS Statistics Version 25 (IBM Corp., Armonk, NY, USA).

## 3. Results

### 3.1. Baseline Clinical Characteristics of the Study Population

**Figure 1:**
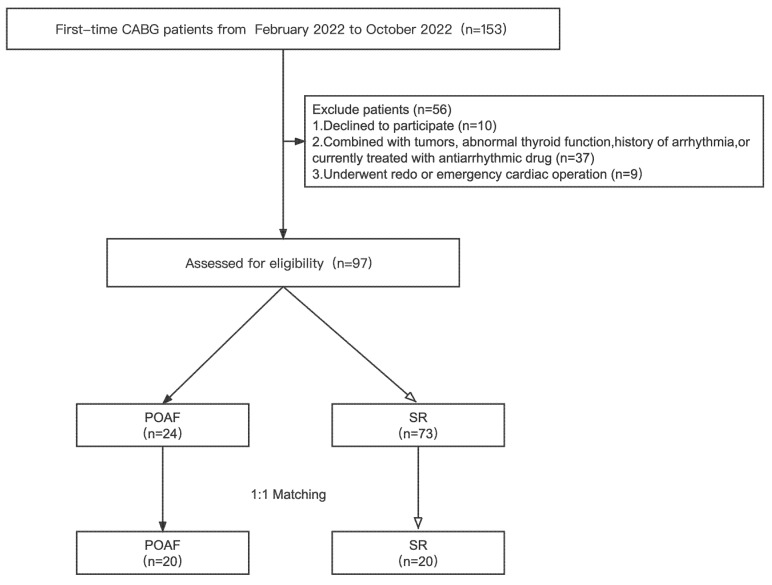
Flow chart of the study. CABG, coronary artery bypass grafting; POAF, postoperative atrial fibrillation; SR, sinus rhythm.

Altogether, 153 patients underwent first-time CABG during the study period. We monitored postoperative heart rhythms in 97 appropriately enrolled patients; 73 (75.26%) patients remained in sinus rhythm (SR) and 24 (24.74%) patients developed POAF (Figure 1). Patients were divided into two groups on the basis of whether they developed POAF (POAF group and SR group, respectively). POAF occurs between days 1–5, on average 2.25 days postoperatively. Among the 24 patients who developed POAF, all the patients were restored to a normal SR by intravenous antiarrhythmic agents.

Patients with POAF were older than those with SR (*p* < 0.05). A history of hypertension was also significantly more frequent in the POAF group than in the SR group. POAF patients had larger left atrial diameters than patients in SR. After a 1:1 matching for the preoperative characteristics, a total of 40 patients were selected for the study. The POAF group was compared to patients in SR with similar preoperative characteristics undergoing CABG (Table 1).

### 3.2. Pericardial Fluid MPO and Serum MPO

**Figure 2:**
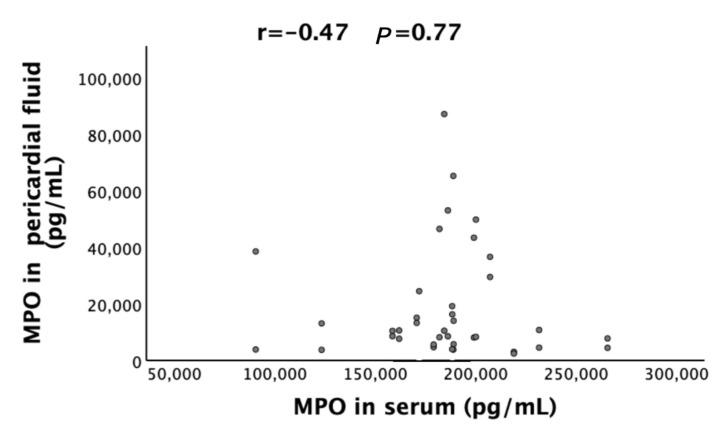
Scatterplots of MPO in serum and in pericardial fluid. r: Spearman correlation coefficients. MPO, myeloperoxidase.

We prospectively measured the levels of MPO in intraoperative pericardial fluid and blood using the human magnetic Luminex assay. In the 40 selected patients (20 patients in the POAF group and 20 patients in the SR group), pericardial fluid MPO levels were significantly lower than serum MPO levels (17801.17 pg/mL (interquartile range 4561.75–23160.05 pg/mL) vs. 184279 pg/mL (170959.25–199858.75 pg/mL), *p* < 0.001). Figure 2 shows the correlation of MPO in serum and in pericardial fluid. No significant correlation was, however, seen between pericardial fluid MPO levels and serum MPO levels (r = −0.47, *p* = 0.77).

### 3.3. Increased Intraoperative Pericardial Fluid MPO Levels in Subjects with POAF

**Figure 3:**
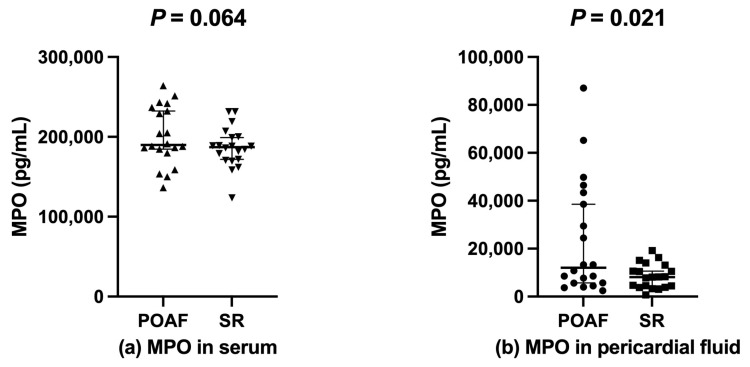
(**a**) 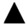: Serum MPO levels in the POAF group; 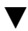: Serum MPO levels in the SR group; (**b**) 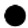: Pericardial fluid MPO levels in the POAF group; 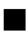: Pericardial fluid MPO levels in the SR group. MPO, myeloperoxidase; POAF, postoperative atrial fibrillation; SR, sinus rhythm.

Intraoperative pericardial fluid MPO levels were significantly higher in the POAF group than in the SR group (*p* = 0.021). There was no difference in the blood MPO between the POAF group and the SR group (*p* = 0.064) (Figure 3).

### 3.4. Association between Intraoperative Pericardial Fluid MPO Levels and POAF

**Figure 4:**
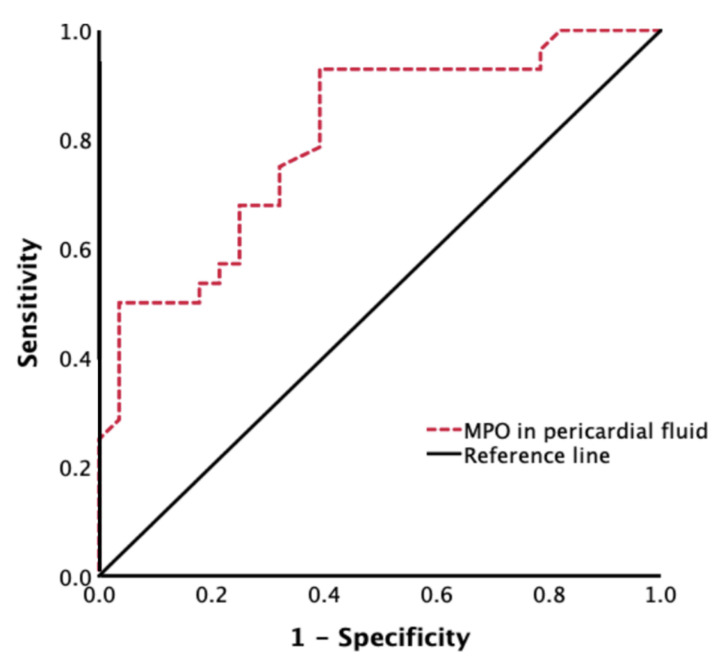
Receiver operator curves using intraoperative pericardial fluid MPO levels for the prediction of POAF. MPO, myeloperoxidase.

Multiple logistic regression analyses were performed to examine whether intraoperative pericardial fluid MPO levels were correlated with POAF. As shown in Table 2, in a multivariable logistic regression model that was adjusted for age, sex, BMI, hypertension, diabetes mellitus, LAD, LVEF, EuroSCORE II, number of grafts, and pericardial fluid MPO levels, the pericardial MPO levels were associated with a higher risk of POAF (odds ratio = 1.016; 95% CI, 1.001–1.031; *p* = 0.031). Based on ROC curve analysis, we determined the optimal threshold value for the optimal meeting point between having the most significant sensitivity and specificity for predicting the occurrence of POAF. The optimal cutoff value of pericardial fluid MPO for identifying POAF was 17,735.50 ng/mL, with a corresponding sensitivity of 96.20% and specificity of 56.50%. The area under the curve (AUC) was 0.805 (Figure 4).

## 4. Discussion

In this study, we used a human magnetic Luminex assay and found that subjects with POAF had significantly elevated intraoperative pericardial fluid MPO levels compared with controls. Intraoperative pericardial fluid MPO levels were highly associated with POAF after CABG. To our knowledge, this is the first study to demonstrate that intraoperative pericardial fluid MPO levels are associated with POAF. These data suggest that the elevation of intraoperative pericardial fluid MPO levels may increase the susceptibility to POAF in patients undergoing CABG.

Factors such as ROS, inflammation, atrial remodeling, preexisting atrial fibrosis, myocardial ischemia, and autonomic nervous system activation are thought to be superimposed on susceptible atrial substrates, making the atrium vulnerable to POAF induction and maintenance [6,22,23]. Other risk factors for the development of POAF, including older age, previous history of atrial fibrillation, sex, decreased left ventricular ejection fraction, left atrial enlargement, valvular heart surgery, hypertension, diabetes, and obesity, have been identified [24,25,26]. However, interventions based on these risk factors and mechanisms do not prevent POAF, indicating that the interindividual susceptibility to POAF remains unclear. One possibility is that patients with a structural substrate before operation and thus, prone to atrial electrical re-entry, are more vulnerable to physiological perturbations that are encountered in the postoperative period. Previous studies have discussed the common pathogenesis between pre-AF and POAF. It is necessary to conduct more in-depth research on the pathogenesis of POAF on the basis of pre-AF to find more targeted prevention and treatment programs.

Previous studies of POAF mostly focused on the plasma of patients. Numerous retrospective studies, meta-analyses, and review papers have been reported identifying POAF risk based on patient risk factors and clinical biomarkers. For example, plasma B-type natriuretic peptide levels, lower plasma phospholipid transfer protein, apolipoprotein-C3, higher cholesteryl ester transfer protein, and glutathione peroxidase 3 levels are linked with POAF [27,28] In those studies, preoperative plasma samples were prospectively collected from patients undergoing CABG procedures. The plasma samples were analyzed using proteomics, metabolomics, and bioinformatics to identify the differential proteins and differential metabolites. POAF prediction based on different studies differs significantly [16,29,30,31]. These studies just explored preoperative plasma in patients with POAF; plasma reflects the overall state of the whole body and has no specificity. The remote but heart-encircling location of pericardial fluid confers unique properties to this biofluid. Therefore, we will use human magnetic Luminex assay technology to identify cytokines related to POAF in pericardial fluid.

Cardiac and surrounding tissues produce many physiologically active substances [32], and the biochemical analysis of pericardial fluid has been proven feasible [33]. Pericardial fluid may have preexisting risk factors before CABG susceptibility to developing POAF. In relation to previous studies on the intraoperative pericardial fluid of patients with POAF, Takeshi Nakamura et al. [34] conducted a prospective study in which they collected pericardial fluid in 42 consecutive patients who were undergoing coronary artery bypass grafting. They measured atrial natriuretic peptide and brain natriuretic peptide levels to determine whether there was an association with the development of POAF. Ultimately, they found that POAF was documented in nine patients (21%), and pericardial fluid brain natriuretic peptide concentration was independently associated with the development of atrial fibrillation after coronary artery bypass grafting. Our data are similar to those of Takeshi Nakamura in that there was a higher level of intraoperative pericardial fluid brain natriuretic peptide in the POAF group vs. the SR group. Our study showed the difference did not reach statistical significance using more samples. Joshua L. et al. [18] also detected 36 intraoperative pericardial fluid cytokines and chemokines, including BNP, and found no significant difference. In our study, intraoperative pericardial fluid brain natriuretic peptide levels were not associated with POAF.

Recent clinical studies relating to pericardial fluid find that several mediators accumulate at a higher level in pericardial fluid than in plasma. Surveys such as those conducted by Joshua L. et al. [18] have shown that mitochondrial DNA in the pericardial fluid was strongly associated with the development of POAF. Yisi Liu et al. [35] provided preliminary evidence of a causal relationship between IL-6 and POAF in a novel nonpaced POAF mouse model. IL-6 is a crucial prerequisite for eliciting profibrotic properties in cardiac myocytes via the pSTAT3 pathway during the early postoperative period, leading to an increased susceptibility to POAF. Accumulating evidence suggests an essential role of inflammatory mechanisms and mediators from pericardial fluid [36]. In conclusion, pericardial fluid analysis appears to be a logical approach for elucidating some cardiac diseases.

In the studies of pre-AF, it has been found that humans with atrial fibrillation had higher plasma concentrations of MPO and a larger MPO burden in right atrial tissue as compared to individuals devoid of atrial fibrillation. In the atria, MPO colocalized with a markedly increased formation of 3-chlorotyrosine. MPO is a crucial prerequisite for the structural remodeling of the myocardium, leading to an increased vulnerability to pre-AF [37]. Yisi Liu et al. [16] explored the local release of MPO into the pericardial space, mainly from damaged atrial tissue, which may be the main source of MPO in postoperative pericardial drainage fluid. Thus, the pericardial MPO concentration is an indicator of the degree of atrial inflammation and atrial MPO activity. However, this study did not measure the concentration of intraoperative pericardial fluid MPO. Our study detected the intraoperative pericardial fluid MPO, and we found that the intraoperative MPO in the pericardial fluid of patients with POAF began to increase compared with that in the SR group (*p* = 0.021). A potential mechanism for the contribution to POAF is the impact of MPO on atrial remodeling and electrical conduction. MPO plays an important role in the tissue damage and the formation of ROS. MPO establishes an intensive pro-oxidative and proinflammatory environment within the pericardium and atrium, leading to early atrial remodeling and disturbing the physiologic function of atrial components. MPO is secreted during inflammation before CABG for patients with ASCVD and causes nitration, chlorination, and oxidation of cardiac tissue. MPO-dependent regulation of matrix metalloproteinase (MMP) activity can increase atrial fibrosis. An animal model of ischemia-related myocardial damage revealed that MPO augments arrhythmogenic left ventricular remodeling and enhanced ventricular post ischemic fibrosis [38]. Thus, MPO contributes to POAF by virtue of its capacity to generate potent ROS and to promote the activity of MMPs.

Dyslipidemia is known to promote atherosclerosis. It is a complex disease and is a major risk factor for adverse cardiovascular events. High levels of low-density lipoprotein cholesterol (LDL-C) and low levels of high-density lipoprotein cholesterol (HDL-C) are associated with myocardial infarction (MI) and stroke. Dyslipidemia is an established risk factor for ASCVD [39]. Previous study reported that total cholesterol (TC) and LDL-C, but not triglycerides (TG) and HDL-C, were associated with the risk for pre-AF [40]. The more lowered the LDL-C in the preoperative period, the more reduced the risk of POAF development. High levels of LDL-C in the preoperative period could be a predictor of POAF [41,42]. In our study, we measured the patients’ TG, TC, LDL-C, HDL-C, lipoprotein-a, and lipid-associated cytokines in serum and pericardial fluid, such as adiponectin, leptin, and fatty acid-binding protein 4 (FABP4). It was found that there was no significant difference between POAF and non-POAF lipid-related laboratory tests and cytokines. In summary, data suggest that the association between blood or pericardial fluid lipid level and risk for POAF is uncertain.

Based on the current results, the MPO cytokines in pericardial fluid are increased and superimposed on the susceptible atrial substrate, making the atrium vulnerable to the induction and maintenance of atrial fibrillation. Pericardial fluid can be collected in cases of open-heart surgery; we suggest incorporating intraoperative pericardial fluid MPO measurements for patients undergoing CABG and facilitating the identification of patients at a higher risk of POAF.

Our data showed that higher intraoperative pericardial fluid MPO levels are linked with POAF in patients undergoing CABG. However, it had several certain limitations. First, the sample size was small; pericardial fluid is difficult to collect; however, it was sufficient to detect differences in pericardial fluid cytokine levels; in the multivariable logistic regression model, the pericardial MPO levels were associated with a higher risk of POAF (odds ratio = 1.016; 95% CI, 1.001–1.031; *p* = 0.031) (the sample size of this study was small and did not meet the requirements of EPV (event per variable)). Therefore, the results may not be robust enough. However, considering that it is difficult to obtain intraoperative pericardial fluid and the results are interpretable, it is still shown. The reliability of this result needs further study. We also 1:1 matched the preoperative data of patients to adjust the configuring factors. Second, our study was not verified at the animal level, and we will conduct animal research in the future to further clarify the role of pericardial fluid cytokines in POAF. Last, in our study, pericardial fluid MPO were lower than serum MPO levels, and subjects with POAF had elevated intraoperative pericardial fluid MPO levels compared with the SR group. We considered, just like the composition of pericardial fluid, MPO in pericardial fluid is not only ultrafiltrate from blood, but may also be secreted from the pericardial cavity; in addition, further research about the specific source of MPO in pericardial fluid is still needed.

## 5. Conclusions

In conclusion, we found that intraoperative pericardial fluid MPO levels were significantly elevated in patients with POAF and were associated with the occurrence of POAF. They could increase the susceptibility to POAF in patients undergoing CABG. It is a powerful direction to prevent POAF in the future. More research is needed to explore its core pathogenesis, and find efficient and safe intervention targets.

## Figures and Tables

**Table 1:** Characteristics of patients with POAF and SR.

	Unmatched			Propensity Score Matched		
	POAF	SR		POAF	SR	
Variable	(n = 24)	(n = 73)	*p* value	(n = 20)	(n = 20)	*p* value
Age (years)	64.07 ± 8.48	61.24 ± 9.06	0.047	63.86 ± 5.10	62.57 ± 7.77	0.468
Sex			0.036			1.000
Male, n (%)	19 (79.17)	60 (82.19)		15 (75.00)	15 (75.00)	
BMI (kg/m^2^)	26.37 ± 3.02	25.73 ± 3.12	0.195	26.51 ± 3.43	26.02 ± 2.42	0.545
Hypertension, n (%)	19 (79.17)	45 (61.64)	0.020	14 (70.00)	12 (60.00)	0.265
Diabetes mellitus, n (%)	13 (54.17)	33 (45.20)	0.239	13 (65.00)	15 (75.00)	0.593
Hyperlipidemia, n (%)	5 (20.83)	19 (26.02)	0.980	6 (30.00)	6 (30.00)	1.000
TG (mmol/L)	1.71 ± 0.83	1.68 ± 0.71	0.824	1.70 ± 0.75	1.70 ± 0.71	0.831
TC (mmol/L)	5.03 ± 1.21	5.05 ± 1.04	0.187	5.03 ± 1.35	5.05 ± 1.01	0.510
HDL-C (mmol/L)	1.01 ± 0.23	0.97 ± 0.30	0.297	1.01 ± 0.23	0.97 ± 0.30	0.639
LDL-C (mmol/L)	3.20 ± 0.85	3.28 ± 0.55	0.248	3.22 ± 0.75	3.24 ± 0.54	0.412
Lp (a) (mmol/L)	73.35 (43.20, 103.41)	87.95 (70.32, 105.59)	0.238	65.47 (43.05, 87.89)	83.84 (48.45, 119.23)	0.373
LVEF (%)	58.72 ± 8.18	59.98 ± 7.79	0.323	60.00 ± 7.56	59.89 ± 7.66	0.958
LAD (mm)	37.27 ± 3.92	35.35 ± 4.24	0.001	37.22 ± 4.34	35.55 ± 3.85	0.274
EuroSCORE II	5.94 ± 2.09	5.41 ± 2.00	0.102	5.86 ± 1.63	5.25 ± 1.48	0.150
Surgery time (h)	4.08 (3.83, 4.33)	3.29 (3.16, 3.42)	0.675	4.08 (3.68, 4.49)	4.16 (3.91, 4.41)	0.412
Number of grafts	4.085 (3.838, 3.322)	4.112 (3.982, 4.243)	0.015	3.61 (3.36, 3.85)	3.61 (3.30, 3.91)	0.864

POAF, postoperative atrial fibrillation; SR, sinus rhythm; BMI, body mass index; TG, triglycerides; TC, total cholesterol; HDL-C, high-density lipoprotein cholesterol; LDL-C, low-density lipoprotein cholesterol; Lp (a), lipoprotein-a; LVEF, left ventricular ejection fraction; LAD, left atrial diameter. Data are displayed as the mean ± standard deviation, median values (+interquartile range), n (%). Differences between groups were analyzed by Fisher’s test, Student’s *t* test, χ^2^ test, or Mann–Whitney U test.

**Table 2:** POAF univariate and multiple logistic regression models.

	Univariate Model			Multiple Model		
	OR	95% CI	*p* Value	OR	95% CI	*p* Value
Age (years)	0.970	0.893–1.052	0.460			
Sex Male	1.00	0.255–3.926	1.000			
BMI (kg/m^2^)	1.059	0.883–1.269	0.538	2.926	1.029–8.320	0.044
Hypertension	1.875	0.618–5.690	0.267			
Diabetes mellitus	0.751	0.263–2.147	0.593	34.489	1.850–643.095	0.017
LVEF (%)	1.002	0.934–1.075	0.957			
LAD	1.535	1.130–5.251	0.525			
EuroSCORE II	0.098	0.011–0.852	0.035			
Number of grafts	2.726	1.102–6.743	0.030			
MPO	1.009	1.001–1.018	0.025	1.016	1.001–1.031	0.031

POAF, postoperative atrial fibrillation; MPO, myeloperoxidase; BMI, body mass index; LVEF, left ventricular ejection fraction; LAD, left atrial diameter; OR, odds ratio; CI, confidence interval.

## References

[1] Perrier S., Meyer N., Hoang Minh T., Announe T., Bentz J., Billaud P., Mommerot A., Mazzucotelli J.P., Kindo M. (2017). Predictors of Atrial Fibrillation After Coronary Artery Bypass Grafting: A Bayesian Analysis. Ann. Thorac. Surg..

[2] Thorén E., Hellgren L., Ståhle E. (2016). High incidence of atrial fibrillation after coronary surgery. Interact. Cardiovasc. Thorac. Surg..

[3] Butt J.H., Olesen J.B., Gundlund A., Kümler T., Olsen P.S., Havers-Borgersen E., Aagaard D.T., Gislason G.H., Torp-Pedersen C., Køber L. (2019). Long-term Thromboembolic Risk in Patients with Postoperative Atrial Fibrillation After Left-Sided Heart Valve Surgery. JAMA Cardiol..

[4] Hindricks G., Potpara T., Dagres N., Arbelo E., Bax J.J., Blomström-Lundqvist C., Boriani G., Castella M., Dan G.A., Dilaveris P.E. (2021). 2020 ESC Guidelines for the diagnosis and management of atrial fibrillation developed in collaboration with the European Association of Cardio-Thoracic Surgery (EACTS). Eur. Heart J..

[5] Gillinov A.M., Bagiella E., Moskowitz A.J., Raiten J.M., Groh M.A., Bowdish M.E., Ailawadi G., Kirkwood K.A., Perrault L.P., Parides M.K. (2016). Rate Control versus Rhythm Control for Atrial Fibrillation after Cardiac Surgery. N. Engl. J. Med..

[6] Dobrev D., Aguilar M., Heijman J., Guichard J.-B., Nattel S. (2019). Postoperative atrial fibrillation: Mechanisms, manifestations and management. Nat. Rev. Cardiol..

[7] Lin M.-H., Kamel H., Singer D.E., Wu Y.-L., Lee M., Ovbiagele B. (2019). Perioperative/Postoperative Atrial Fibrillation and Risk of Subsequent Stroke and/or Mortality. Stroke.

[8] Eikelboom R., Sanjanwala R., Le M.L., Yamashita M.H., Arora R.C. (2021). Post-operative atrial fibrillation after cardiac surgery: A systematic review and meta-analysis. Ann. Thorac. Surg..

[9] Hoit B.D. (2017). Pathophysiology of the Pericardium. Prog. Cardiovasc. Dis..

[10] Stewart D.J., Cernacek P., Costello K.B., Rouleau J.L. (1992). Elevated endothelin-1 in heart failure and loss of normal response to postural change. Circulation.

[11] Hasdai D., Barak V., Leibovitz E., Herz I., Sclarovsky S., Eldar M., Scheinowitz M. (1997). Serum basic fibroblast growth factor levels in patients with ischemic heart disease. Int. J. Cardiol..

[12] Winterbourn C.C., Kettle A.J., Hampton M.B. (2016). Reactive Oxygen Species and Neutrophil Function. Annu. Rev. Biochem..

[13] Fontes M.L., Mathew J.P., Rinder H.M., Zelterman D., Smith B.R., Rinder C.S. (2005). Atrial Fibrillation After Cardiac Surgery/Cardiopulmonary Bypass Is Associated with Monocyte Activation. Anesth. Analg..

[14] Ndrepepa G. (2019). Myeloperoxidase—A bridge linking inflammation and oxidative stress with cardiovascular disease. Clin. Chim. Acta.

[15] Baldus S., Heeschen C., Meinertz T., Zeiher A.M., Eiserich J.P., Münzel T., Simoons M.L., Hamm C.W. (2003). Myeloperoxidase Serum Levels Predict Risk in Patients with Acute Coronary Syndromes. Circulation.

[16] Liu Y., Yu M., Wu Y., Wu F., Feng X., Zhao H. (2021). Myeloperoxidase in the pericardial fluid improves the performance of prediction rules for postoperative atrial fibrillation. J. Thorac. Cardiovasc. Surg..

[17] Leligdowicz A., Conroy A.L., Hawkes M., Zhong K., Lebovic G., Matthay M.A., Kain K.C. (2017). Validation of two multiplex platforms to quantify circulating markers of inflammation and endothelial injury in severe infection. PLoS ONE.

[18] Manghelli J.L., Kelly M.O., Carter D.I., Gauthier J.M., Scozzi D., Lancaster T.S., MacGregor R.M., Khiabani A.J., Schuessler R.B., Gelman A.E. (2021). Pericardial Mitochondrial DNA Levels Are Associated with Atrial Fibrillation After Cardiac Surgery. Ann. Thorac. Surg..

[19] Shenje J., Lai R.P., Ross I.L., Mayosi B.M., Wilkinson R.J., Ntsekhe M., Wilkinson K.A. (2017). Effect of prednisolone on inflammatory markers in pericardial tuberculosis: A pilot study. IJC Heart Vasc..

[20] Kirchhof P., Benussi S., Kotecha D., Ahlsson A., Atar D., Casadei B., Castella M., Diener H.C., Heidbuchel H., Hendriks J. (2016). 2016 ESC Guidelines for the management of atrial fibrillation developed in collaboration with EACTS. Eur. Heart J..

[21] Lonjon G., Porcher R., Ergina P., Fouet M., Boutron I. (2017). Potential Pitfalls of Reporting and Bias in Observational Studies with Propensity Score Analysis Assessing a Surgical Procedure: A Methodological Systematic Review. Ann Surg..

[22] Borger M.A., Mansour M.C., Levine R.A. (2019). Atrial Fibrillation and Mitral Valve Prolapse: Time to Intervene?. J. Am. Coll. Cardiol..

[23] Kim Y.M., Kattach H., Ratnatunga C., Pillai R., Channon K.M., Casadei B. (2008). Association of Atrial Nicotinamide Adenine Dinucleotide Phosphate Oxidase Activity With the Development of Atrial Fibrillation After Cardiac Surgery. J. Am. Coll. Cardiol..

[24] Mathew J.P., Fontes M.L., Tudor I.C., Ramsay J., Duke P., Mazer C.D., Barash P.G., Hsu P.H., Mangano D.T. (2004). A Multicenter Risk Index for Atrial Fibrillation After Cardiac Surgery. JAMA.

[25] Banach M., Rysz J., Drozdz J.A., Okonski P., Misztal M., Barylski M., Irzmanski R., Zaslonka J. (2006). Risk factors of atrial fibrillation following coronary artery bypass grafting: A preliminary report. Circ. J..

[26] Echahidi N., Mohty D., Pibarot P., Després J.-P., O’Hara G., Champagne J., Philippon F., Daleau P., Voisine P., Mathieu P. (2007). Obesity and Metabolic Syndrome Are Independent Risk Factors for Atrial Fibrillation After Coronary Artery Bypass Graft Surgery. Circulation.

[27] Evogiatzidis K., Zarogiannis S.G., Aidonidis I., Solenov E.I., Emolyvdas P.-A., Gourgoulianis K.I., Ehatzoglou C. (2015). Physiology of pericardial fluid production and drainage. Front. Physiol..

[28] Burgess L. (2004). Biochemical analysis of pleural, peritoneal and pericardial effusions. Clin. Chim. Acta.

[29] Nakamura T., Azuma A., Sawada T., Sakamoto K., Yamano T., Yaku H., Matsubara H. (2007). Brain natriuretic peptide concentration in pericardial fluid is independently associated with atrial fibrillation after off-pump coronary artery bypass surgery. Coron. Artery Dis..

[30] Liu Y., Wu F., Wu Y., Elliott M., Zhou W., Deng Y., Ren D., Zhao H. (2021). Mechanism of IL-6-related spontaneous atrial fibrillation after coronary artery grafting surgery: IL-6 knockout mouse study and human observation. Transl. Res..

[31] Fujita M., Komeda M., Hasegawa K., Kihara Y., Nohara R., Sasayama S. (2001). Pericardial fluid as a new material for clinical heart research. Int. J. Cardiol..

[32] Wazni O.M., Martin D.O., Marrouche N.F., Latif A.A., Ziada K., Shaaraoui M., Almahameed S., Schweikert R.A., Saliba W.I., Gillinov A.M. (2004). Plasma B-Type Natriuretic Peptide Levels Predict Postoperative Atrial Fibrillation in Patients Undergoing Cardiac Surgery. Circulation.

[33] Li X.-Y., Hou H.-T., Chen H.-X., Liu X.-C., Wang J., Yang Q., He G.-W. (2021). Preoperative plasma biomarkers associated with atrial fibrillation after coronary artery bypass surgery. J. Thorac. Cardiovasc. Surg..

[34] Khan M.S., Yamashita K., Sharma V., Ranjan R., Selzman C.H., Dosdall D.J. (2020). Perioperative Biomarkers Predicting Postoperative Atrial Fibrillation Risk After Coronary Artery Bypass Grafting: A Narrative Review. J. Cardiothorac. Vasc. Anesth..

[35] Anatoĺevna R.O., Veniaminovich F.O., Mikhaylovich K.S. (2016). Predictors of new-onset atrial fibrillation in elderly patients with coronary artery disease after coronary artery bypass graft. J. Geriatr. Cardiol..

[36] Paschalis A., Tousoulis D., Demosthenous M., Antonopoulos A., Papaioannou S., Miliou A., Koumallos N., Antoniades C., Stefanadis C. (2014). Pre-operative inflammation and post-operative atrial fibrillation in coronary artery bypass surgery. Int. J. Cardiol..

[37] Rudolph V., Andrié R.P., Rudolph T.K., Friedrichs K., Klinke A., Hirsch-Hoffmann B., Schwoerer A.P., Lau D., Fu X., Klingel K. (2010). Myeloperoxidase acts as a profibrotic mediator of atrial fibrillation. Nat. Med..

[38] Aratani Y. (2018). Myeloperoxidase: Its role for host defense, inflammation, and neutrophil function. Arch. Biochem. Biophys..

[39] Mach F., Baigent C., Catapano A.L., Koskinas K.C., Casula M., Badimon L., Chapman M.J., De Backer G.G., Delgado V., Ference B.A. (2019). 2019 ESC/EAS guidelines for the management of dyslipidaemias: Lipid modification to reduce cardiovascular risk. Atherosclerosis.

[40] Lopez F.L., Agarwal S.K., Maclehose R.F., Soliman E.Z., Sharrett A.R., Huxley R.R., Konety S., Ballantyne C.M., Alonso A. (2012). Blood lipid levels, lipid-lowering medications, and the incidence. of atrial fibrillation: The atherosclerosis risk in communities study. Circ. Arrhythm. Electrophysiol..

[41] Aydin M., Susam I., Kilicaslan B., Dereli M., Sacar M., Ozdogan O. (2014). Serum cholesterol levels and postoperative atrial fibrillation. J. Cardiothorac. Surg..

[42] Tadic M., Ivanovic B., Zivkovic N. (2011). Predictors of atrial fibrillation following coronary artery bypass surgery. Med. Sci. Monit..

